# The Impact of Using Different Doses of Biomass Ash on Some Physical Properties of Podzolic Soil under the Cultivation of Winter Oilseed Rape

**DOI:** 10.3390/ijerph19116693

**Published:** 2022-05-30

**Authors:** Jadwiga Stanek-Tarkowska, Ewa Antonina Czyż, Miłosz Pastuszczak, Karol Skrobacz

**Affiliations:** Department of Soil Science, Environmental Chemistry and Hydrology, University of Rzeszow, Zelwerowicza 8B, 35-601 Rzeszów, Poland; eczyz@ur.edu.pl (E.A.C.); miloszp@dokt.ur.edu.pl (M.P.); kskrobacz@ur.edu.pl (K.S.)

**Keywords:** soil moisture, soil bulk density, penetration resistance, readily dispersible clay, biomass ash

## Abstract

This two-year study was focused on the effect of the application of different biomass ash doses on selected soil physical properties, i.e., soil moisture (SM), bulk density (BD), penetration resistance (PR), and soil stability in water measured by the content of readily dispersible clay (RDC), following control and mineral NPK fertilization in the cultivation of winter oilseed rape (*Brassica napus* L. var. napus). A one-factor field experiment conducted on podzolic soil (control, NPK, 100, 200, 300, 400, 500 kg K_2_O·ha^−1^) showed that the use of biomass combustion ash significantly improved soil moisture at all depths and variants, and especially at a depth of 30–35 cm in the 500 kg·ha^−1^ variant, i.e., by 2.99% *v*/*v*, compared to NPK. In turn, the moisture content in the 30–35 cm layer increased by 3.19% *v*/*v* in all variants in both years compared to the control. In 2020 and 2021, bulk density in the 0–5 cm layer treated with a dose of 500 kg·ha^−1^ exhibited a positive 0.15 and 0.12 Mg·m^−3^ decrease, respectively, compared to the control. In both years, the BD values in the 30–35 cm layer were reduced by 0.14 and 0.16 Mg·m^−3^ compared to the control. The PR values decreased in the treatments with doses of 300, 400, and 500 kg·ha^−1^, especially in 2021. The RDC content was found to decline in both years, i.e., 2020 and 2021, upon the application of even the lowest dose (100 kg·ha^−1^) in all the analysed layers. The reduction in the RDC content, especially in the 0–5 cm layer, is very important for soil structure stability and to protect the soil environment. This layer is most susceptible to crusting, which results in poor aeration and weak plant emergence during drought and/or periods of excessive moisture. It may also increase surface runoff and intensify soil erosion processes.

## 1. Introduction

Wood ash, i.e., the product of the complete combustion of woody materials, can be used in plant nutrition and nutrient cycling in agriculture [1,2]. Since it contains appropriate proportions of various macro- and micro- nutrients and a mixture of oxides, hydroxides, carbonates, and silicates [3,4], wood ash is a potentially excellent soil fertilizer which is gaining wide acceptance as an alternative to chemical fertilizers. As a potential contributor to the circular economy system, wood ash is one of the most sustainable options available to meet the increasing demand for bioenergy and improved soil fertility [5,6]. Although biomass ash has potential beneficial applications in soil or construction products, such residues are still largely landfilled. This unsustainable practice is a result of regulatory barriers and concerns about the potential leaching of pollutants into soil ecosystems. To facilitate biomass ash recycling and sustainable biomass conversion, it is important to assess the benefits and risks of the reuse of this type of ash. The regulations and criteria for the use of biomass ash in forestry, agriculture, or the construction industry vary among European countries.

Research on the application of biomass ash on crop soils was conducted by the authors of [2]. Other studies have indicated that wood ash, due to its calcifying effect, can also replace calcium products and counteract soil acidification [7,8,9,10]. Bang-Andereasen et al. [11,12] described the beneficial effect of using wood ash in their works. Additionally, research by Brunner et al. (2004) [13] focused on the beneficial effects of wood ash on forest soils.

Biomass combustion ash is increasingly being used in agriculture as a fertilizer due to its beneficial effects on soil, i.e., improvement of biological and chemical properties; however, few studies have been published on the impact of ash on soil physical properties. Properties such as soil moisture content (SM), soil bulk density (BD), penetration resistance (PR), readily dispersible clay (RDC), soil stability in water, etc., are among of the most important parameters [14,15,16,17,18,19,20,21,22].

In their study, Bonfim-Silva et al. [23] demonstrated that soil compaction is a limiting factor in agricultural production. In addition to triggering physical changes, it hinders of root penetration and reduces soil macroporosity, thereby affecting dynamic processes related to the availability and infiltration of water, as well as the flow of gases in the soil profile [24,25,26]. The compaction of agricultural soils has been investigated by many soil scientists and farmers, as this phenomenon caused by heavy tractor and agricultural machinery traffic in fields has been found to reduce the yields of most agriculture crops worldwide. Excessive soil compaction is a physical form of soil degradation that alters the soil structure and limits water and air infiltration. The consequences of soil compaction are still likely underappreciated [27]. It is estimated that approximately 68 million hectares of land worldwide is affected by soil compaction as a result of wheel traffic. Compaction is responsible for soil degradation in Europe (33 million ha), Africa (18 million ha), Asia (10 million ha), Australia (4 million ha), and some areas of North America [28,29].

Another important parameter is bulk density (BD), which is an indicator of soil compaction. BD has an impact on infiltration, rooting depth, available water capacity, porosity, soil aeration, the availability of nutrients for plant use, and the activity of soil microorganisms, all of which affect key soil processes. Bulk density (BD) is not an intrinsic property of soil, but rather, depends on external conditions, i.e., various natural and anthropogenic factors [30]. It is a major factor in soil compaction and changes almost immediately after reduction tillage-induced soil disturbance. Intensive work of heavy machinery and tools in agricultural land leads to soil compaction, which may increase bulk density and limit water and air transport in the soil [21,31]. Soil bulk density plays a fundamental role in determining the physical condition of the soil and its ability to support plant growth and store soil organic carbon. Soils with lower bulk density have a good structure, a larger surface area, and a greater capacity for the retention of water, nutrients, and organic carbon. Bulk density also regulates the movement of water and gases in the soil and its interface with the environment. This is essential for understanding the changes induced by anthropogenic disturbances such as land use and agricultural management. Bulk density is an inherent property of soil and depends on mineral and organic composition and water contents [21,32]. Additionally, it may change as a result of erosion or anthropopressure-related interactions, e.g., the use and management of agricultural land. Due to the complex dynamics of these interactions, bulk density can vary significantly over short distances across the landscape and at depth. Additionally, it can change with time and seasons due to land management practices such as tillage.

Another parameter, i.e., the content of readily dispersible clay (RDC), is closely related to soil bulk density (BD) and penetration resistance. According to Gajda et al. [32], RDC parameters are dynamic, and its content in the soil is determined by many factors, e.g., soil wetting and drying and the soil organic matter [21]. RDC is regarded as an indicator of soil structure in water and can be used to monitor possible modifications indicating the improvement or deterioration of soil structure [33,34]. Various studies [14,32,35,36,37,38] have indicated problems associated with dispersed clay. A high RDC content can cause soil crusting, reduce water infiltration, and increase surface runoff. Therefore, soil susceptibility to destruction is an extremely important issue for environmental protection and agricultural production [35].

The aim of this study was to determine the effect of the application of different biomass ash doses on soil properties, such as moisture content, bulk density, penetration resistance, and soil stability in water measured by the content of readily dispersible clay, versus NPK mineral fertilization and control variants for the cultivation of winter oilseed rape.

The investigations sought to verify the hypothesis that the use of different doses of biomass ash would have a positive effect on soil by improving its physical properties.

## 2. Materials and Methods

Field experiments were performed on a property on podzolic soil under *Brassica napus* L. cultivar Mandril (*Syngenta*) in 2018 (autumn) in Korzenica, Jaroslaw county (Podkarpackie voivodeship in Poland) GPS coordinates: 50.02′0.238″ N, 20.22.55′0.198″ E; 50.02′0.198″ N, 20.55′0.124″ E. The podzolic soil had the particle size distribution of silty loam (Table 1). The granulometric subgroups followed the recommendations of the United States Department of Agriculture (USDA) [39].

As a single-factor field experiment, the randomized block method (each block of approximately 162 m^2^) was applied in triplicate. The dose of biomass combustion ash fertilizer (*Salix viminalis* L willow) was the experimental variable. The obtained results were compared with a control soil—i.e., without fertilization and with soil subjected exclusively to NPK mineral fertilization. The biomass ash doses were balanced to the amount of potassium introduced into the soil. All the variants were treated with constant mineral fertilization with nitrogen (81.3 kg N ha^−1^) and phosphorus (34 kg P ha^−1^). Variants of the experiment were as follows:

- Control—no K_2_O fertilization;
- NPK—K_2_O in mineral fertilizers (127 kg K_2_O ha^−^^1^);
- W1—100 kg K_2_O ha^−^^1^ in ash;
- W2—200 kg K_2_O ha^−^^1^ in ash;
- W3—300 kg K_2_O ha^−^^1^ in ash;
- W4—400 kg K_2_O ha^−^^1^ in ash;
- W5—500 kg K_2_O ha^−^^1^ in ash.

In autumn, during the season preceding the beginning of the experiment, calculated doses of fertilizer were applied. The doses and dates of application are presented in Table 2.

The composition of the biomass ash used in the experiment to fertilize winter oilseed rape on podzolic soil is presented in Table 3.

Soil samples were collected in 2020 to 2021 before the oilseed rape harvest. Depending on the parameter being determined, soil samples were collected differently without and with disturbing the soil structure. The samples were collected in 100 cm cylinders (for determination of soil moisture and bulk density) or in plastic bags (for determination of soil stability in water, measured by readily dispersible clay (RDC) content).

For the determination of SM and BD, samples were collected in 10 replicates from the 0–5 and 30–35 cm layers into cylinders with a volume of 100 cm^3^. Soil moisture and bulk density were measured in the laboratory by weighing the soil samples before and after drying at 105 °C in an oven for 48 h.

The soil moisture, SM, values were then calculated as a % volume using the following equation:

(1) $$\mathrm{Soil}\;\mathrm{moisture},\;\mathrm{SM}=\frac{\left(m_{w}+t\right)-\left(m_{d}+t\right)}{100}$$

where SM—volumetric soil moisture (% *v*/*v*)

*m_w_*—wet soil mass (g)
*m_d_*—weights of oven-dry soils
*t*—tare of cylinder (g)
100—volume of the cylindrical core.

Bulk density (BD) was determined after drying the core samples in an oven at 105 °C for 48 h and calculated as:

(2) $$\mathrm{Bulk}\;\mathrm{density},\;\mathrm{BD}=\frac{m_{d}}{100}$$

where BD—dry bulk density (Mg·m^−3^)

*m_d_*—weights of oven-dry soils,
100—volume of the cylindrical core.

A total of 840 samples were measure for bulk density (2 depths × 10 replications × 3 plots × 2 years × 7 treatment) and 840 samples for gravimetric soil moisture (2 depths × 10 replications × 3 plots × 2 years × 7 treatment).

Soil penetration resistance, *PR* (Pa or MPa or N·m^−2^), is defined as the force, *P* (N), required for cone penetration divided by the area *A* (m^2^) of the widest part of the cone:

(3) $$\mathrm{Penetration}\;\mathrm{resistance},\;\mathrm{PR}=\frac{P}{A}$$

Soil penetration resistance, PR was measured using an automatic penetrologger (type 06.15.SA Eijkelkamp Agrisearch Equipment, Giesbeek, The Netherlands) with a 1 cm^2^ diameter and a 30° angle cone. Penetration resistance was recorded automatically at 1 cm intervals. Since the penetration resistance is variable, ten parallel measurements were performed for each experimental variant. The median was calculated from each measurement. The total number of penetrations was 420 (7 treatments × 10 replicates × 3 plots × 2 years). In total, 420 soil compactness measurements to a depth of 80 cm were analysed.

Soil samples for the determination of readily dispersible clay (RDC) were collected at the following depths: 0–5, 5–10, 10–15, 15–12, 20–25, 25–30 and 30–35 cm. This parameter was determined using the method described by Czyż and Dexter [41]. Soil stability in water was measured in terms of readily dispersible clay (RDC) content expressed in g per 100 g of soil, using a Hach 2100 AN ratio turbidimeter [41]. A total of 294 soil samples were collected in triplicate for each depth, and then analysed.

Meteorological data for 2019–2021 were provided by the University of Rzeszow meteorological station.

The obtained results were analysed using STATISTICA 13.3 software (StatSoft, Tulsa, OK, USA). One-way analysis of variance (ANOVA) was performed to identify homogeneous groups (*p* < 0.03) using Tukey HSD multiple comparisons test.

## 3. Results and Discussion

### 3.1. Meteorogical Condition

**Figure 1:**
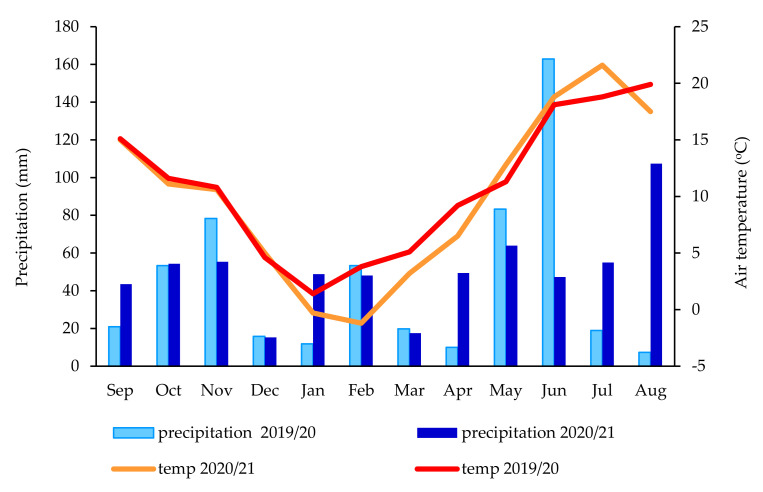
Weather conditions during cultivation of winter oilseed rape in 2019–2021 provided from the Meteorological Station of the University of Rzeszow.

The total precipitation in the studied years, i.e., 2019–2021, varied (Figure 1): in 2019/2020, it was 535.6 mm during the *Brassica napus* L. growing season, while in 2020/2021 it was higher at 605.9 mm. However, during the soil sampling month (August), the difference in total precipitation was 100.1 mm. There was 7.3 mm of precipitation in 2020 and 107.4 mm in 2021. The average air temperature during the 2019/2020 growing season was 10.8 °C; in 2020/2021, this was 0.7 °C lower than in the first year, averaging 10.1 °C.

### 3.2. Soil Moisture

The most important water properties of soils include current moisture, water capacity, plant–available water retention, and water conductivity [42,43]. Current soil moisture reflects the water content at the time of soil sampling. Sufficient soil water is essential for plant life and soil microorganisms. Current soil moisture is the most dynamic property in the soil; changes thereof are mainly induced by precipitation, transpiration, and evaporation of water from the soil surface, and cultivation treatments [44]. Especially in dry periods, this factor is dependent on the soil granulometric composition, soil structure, and compaction. Soil moisture content is closely correlated to the type of soil, i.e., its granulometric composition, water retention, meteorological conditions (precipitation and temperature) agrotechnical treatments, and type of crops [33,45,46].

**Figure 2:**
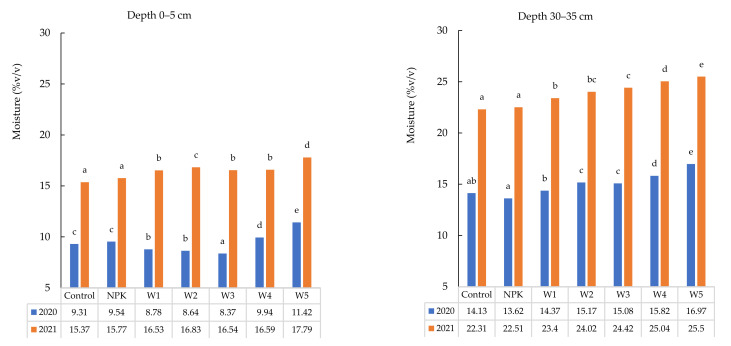
Variability of soil moisture in podzolic soil under winter oilseed rape cultivation in different variants of biomass ash fertilization. Different lowercase letters indicate significant differences between variant by Tukey’s HSD post hoc test (*p* > 0.03).

The present experiment demonstrated (Figure 2) that soil moisture was influenced by the fertilization type, i.e., the content of soil water depended on the dose of ash applied in the experimental variants. In 2020, the difference between the NPK and W5 variants (500 kg·ha^−1^) in the 0–5 cm layer was 1.88% *v*/*v* in favour of ash application. In 2021, the soil moisture in the same layer was 2.02% *v*/*v* higher. A similar increase in moisture was recorded in all variants and layers; however, the 30–35 cm layer exhibited a 3.35% *v*/*v* increase in W5 versus NPK in 2020. In 2021, higher water content was recorded at all layers and variants, with a particularly large increase (by 2.99% *v*/*v*) in W5 versus NPK at the depth of 30–35 cm. The experiment showed that increasing biomass ash dose resulted in a significant increase in the water content in the soil in the 0–5 and 30–35 cm layers. Similarly, Pereira et al. [47] reported a beneficial effect of biomass combustion ash on soil water content. The comparison of the soil moisture content between the different ash fertilization variants and the control revealed an increase in the value of this parameter in W4 and W5 in 0–5 cm layer. In turn, the 30–35 cm layer was characterized by an increase in the moisture content in all variants in both years.

### 3.3. Bulk Density

**Figure 3:**
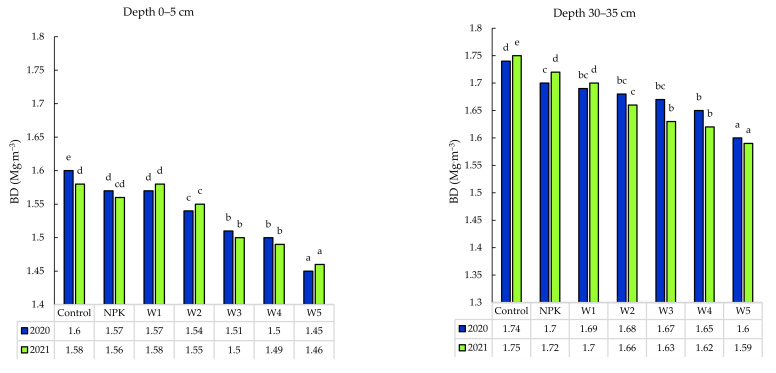
Variability in bulk density (BD) of podzolic soil under winter oilseed rape under different biomass ash fertilization variants. Different lowercase letters indicate significant differences between variant by Tukey’s HSD post hoc test (*p* > 0.03).

Bulk density (BD) is the most frequently used parameter to assess soil compaction. It is also regarded as an indirect indicator of soil structure, penetration resistance, porosity, aeration, and soil capacity to store and transport soil water [48]. An increase of soil density causes a proportional increase in mechanical resistance, decrease in total porosity, content of macropores, water conductivity air permeability, and deterioration of oxygenation and biological properties of soils [22,49,50,51]. The thermal conductivity and heat capacity of soil increase with the increase in the compaction and tighter packing of soil particles. Excessive soil compaction exerts an adverse effect on the plant root system. It reduces the length and depth of the root system and the distance between roots [51,52,53,54]. Highly compacted soil is characterized by uneven spatial distribution of roots or even absence of roots in some parts. Our two-year study (Figure 3) demonstrated that the application of biomass ash reduced BD values which is beneficial for the status of the soil and plant and, statistical analysis showed significant differences. The bulk density in the 0–5 cm layer decreased by 0.15 Mg·m^−3^ in 2020 and by 0.12 Mg·m^−3^ in 2021, compared to the control. There was also a downward trend in the BD value by 0.14 Mg·m^−3^ in the 30–35 cm layer in 2020, compared to the control. In the second year of the experiment (2021), the value of this parameters decreased by 0.16 Mg·m^−3^, compared to the control. Moreover, a downward trend was found in the variants of fertilization, especially in W4 and W5, compared to the NPK treatment in both years. A study conducted by Bonfilm-Silva et al. [23] unequivocally supports the present results, which indicated that the ash fertilization with the combustion biomass ash caused a decrease in the soil BD value in both years. This finding is important, as it suggests a possible means of increasing the soil water holding capacity.

### 3.4. Penetration Resistance

**Figure 4:**
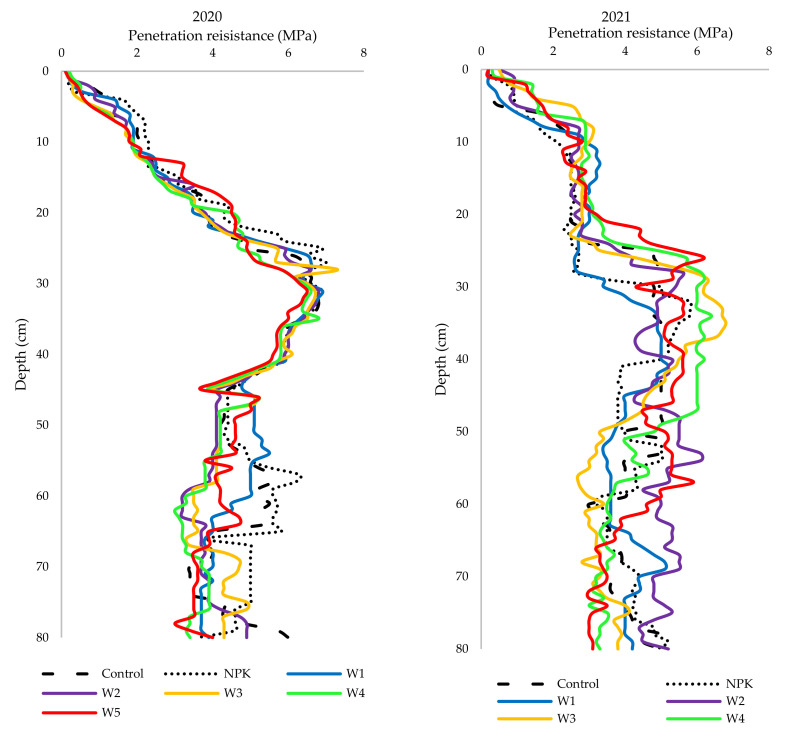
Variability of penetration resistance of podzolic soil under winter oilseed rape cultivation in different variants of biomass ash fertilization.

Soil compaction can be a natural phenomenon [55], associated with freezing and drying, or an artificial phenomenon induced by mechanical operations [56]. It may be defined as “the process of rearrangement of the soil grains to reduce voids and bring the grains closer together, thereby increasing the bulk density”. Soil compaction is also accompanied by the modification of other physical, chemical, and biological parameters [26]. The rapid compaction of arable land caused by the use of heavy agricultural machinery is inevitable. Our two-year experiment with the application of biomass combustion ash indicated that ash doses ranging from 200 to 500 kg·ha^−1^ had a beneficial effect, i.e., they reduced the compactness of the podzolic soil, especially at a depth of 30–35 cm in 2021 (Figure 4).

Over-compaction is a form of physical deterioration of soils and is a worldwide problem. Many researchers have investigated the effects of various farming systems on soil compaction, but to date, no comprehensive investigations have been published of the use of unconventional fertilizers, e.g., biomass combustion products, on this soil parameter. The present study shows that the application of ash reduced the penetration resistance in the podzolic soil in both study years.

### 3.5. Readily Dispersible Clay

Clay is a key component of any soil. When it is compacted, its particle are bound together, constituting a component for other soil particles. Soil is stable when it is wet or exposed to the impact of water. Stable soils have low RDC content. The clay dispersion phenomenon includes the repellence and movement of clay particles as a suspension between larger soil particles. Therefore, a high content of readily dispersible clay in soil triggers two phenomena: weakness and leaching of wet soils and excessive hardness and cementation of dry soils [35].

**Figure 5:**
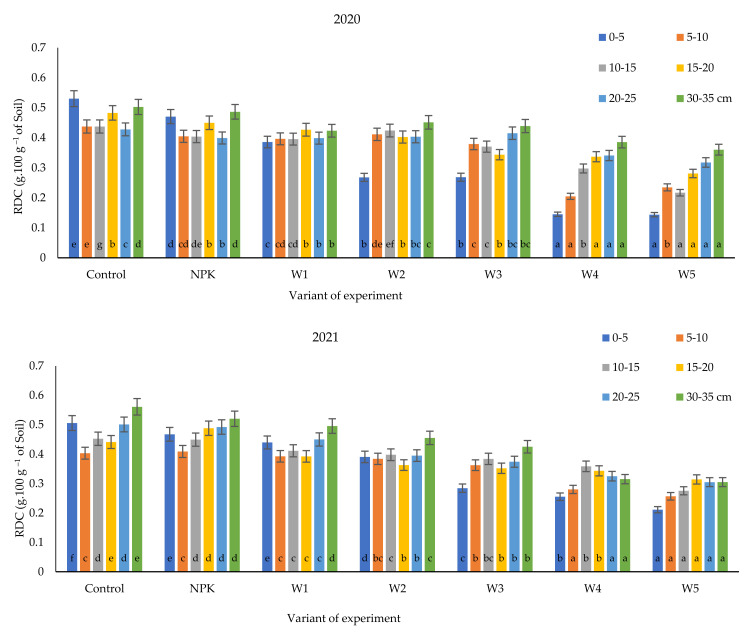
Variability of RDC content in the soil profile of a podzolic soil under winter oilseed rape cultivation under different biomass ash fertilization variants. Different lowercase letters indicate significant differences between variant by Tukey’s HSD post hoc test (*p* > 0.05).

Our study (Figure 5) shows that the positive reduction in RDC content in both years i.e., 2020 and 2021, was significant, not only at the lowest ash dose of 100 kg·ha^−1^, but also at doses of 400 and 500 kg·ha^−1^ at all investigated depths. An equally beneficial reduction in RDC content was recorded in the 0–5 cm soil layer, which is most susceptible to crusting or erosion [22].

The ability to prevent an increase in the content of readily dispersible clay in soils is important, as this parameter is invoved in global soil erosion phenomena. As shown in the present study, the use of doses of biomass ash ranging from 100 to 500 kg·ha^−1^ considerably improves the physical properties of podzolic soil.

## 4. Conclusions

Most investigations on the use of unconventional fertilizers (biomass or sewage sludge) have been focused on the influence of these materials on the chemical properties of soil, whereas the issue of soil physical properties has been poorly explored.

The present study shows that biomass ash has a positive effect on the physical properties of soil, i.e., soil moisture, bulk density, penetration resistance, and reduction of readily dispersible clay content. These results allow us to conclude that the use of biomass ash, especially in high doses, reduces soil bulk density. We also recorded lower values of penetration resistance in podzolic soil at doses of 300–500 kg·ha^−1^ of biomass ash. The application of ash significantly improved soil moisture and reduced the content of readily dispersible clay, thereby increasing soil stability in water, which is important for the prevention of soil erosion.

In general, the use of biomass ash improved all the analysed physical properties of podzolic soil. Biomass combustion ash may become an alternative fertilizer that is capable of improving the physical properties of soil, which are currently underestimated and poorly studied.

## Figures and Tables

**Table 1:** Particle size distribution of the soil used in the experimental field.

Depth (cm)	Sand 0.05–2.0 mm	Silt 0.002–0.05 mm	Clay <0.002 mm
0–5	50	46	4
5–10	52	43	5
10–15	50	47	3
15–20	46	51	3
20–25	52	45	3
30–35	51	46	3

**Table 2:** Fertilizers used in the field experiment on podzolic soil in 2018–2021 [40].

Fertilizer	Content of Pure Component in 100 kg of Fertilizer	Dose (kg/L per 1 ha)		Fertilization Term
		Fertilizer	Pure Component	
Ash from biomass combustion	1.63% P (3.73 kg P); 19.4% K (23.37 kg K); 4.96% Mg (8.22 kg Mg)	Varies according to experiment variant		30.08.2018 29.08.2019 25.08.2020
Monoammonium phosphate (MAP) NH_4_H_2_PO_4_ (12% N-NH_4_, 52% P_2_O_5_, 22.7% P)	22.7 kg P	150	34	30.08.2018 (all plots) 29.08.2019 (all plots) 25.08.2020 (all plots)
	12 kg N		18	
Potassium salt (60%)	60 kg K	175	105	30.08.2018 (NPK plots only) 29.08.2019 (NPK plots only) 28.08.2020 (NPK plots only)
RSM^®^ 32% N (ammonium urea nitrate, water solution, density 1.32 kg/dcm^3^)	42.2 kg N (32 × 1.32)	150	63.3	4.03.2019 10.03.2020 15.03.2021

**Table 3:** Composition of biomass ash used in the experiment for fertilization of winter oilseed rape on podzolic soil.

pH H_2_O	EC µS·cm^−^^1^	Ca (mg kg^−^^1^)	K (mg kg^−^^1^)	Na (mg kg^−^^1^)	P (mg kg^−^^1^)
12.82	8.81	145.081	129.617	1452	9244

## Data Availability

The data presented in this study are available in this article.
